# Passivity-based control of an omnidirectional mobile robot

**DOI:** 10.1186/s40638-016-0037-z

**Published:** 2016-07-07

**Authors:** Chao Ren, Yi Sun, Shugen Ma

**Affiliations:** School of Electrical Engineering and Automation, Tianjin University, 92 WeijinRoad, Tianjin, 300072 China; Department of Robotics, Ritsumeikan University, 1-1-1 Noji Higashi, Kusatsu, Shiga 525-8577 Japan

**Keywords:** Passivity, Dynamic control, Omnidirectional mobile robot

## Abstract

This paper studies passivity-based trajectory tracking control of an omnidirectional mobile robot. The proposed control design is simple to be implemented in practice, because of an effective exploitation of the structure of robot dynamics. First, the passivity property of the prototype robot is analyzed. Then the control system is designed based on the energy shaping plus damping approach. We find that the prototype robot itself has enough damping forces. As a result, only energy shaping is needed in our proposed controller, while the damping injection is unnecessary for our robot. In other words, the disadvantages of differential feedback, such as amplifying the measurement noise, can be avoided. Globally asymptotic stability is guaranteed. Both simulations and experimental results show the effectiveness of the proposed control design.

## Background

Omnidirectional mobile robots (OMRs) are becoming increasingly popular in many applications. OMRs have the ability to move simultaneously and independently in translational and rotational motion. Therefore, they are especially useful in environments congested with static and dynamic obstacles and narrow aisles, such as hospitals, warehouses, residential homes, and sheltered workshops for disabled people.

In the literature, many studies have been conducted in the dynamic model-based control design for OMRs. In [1], a feedback linearization approach, i.e., resolved acceleration control, was applied to an OMR with lateral orthogonal-wheels. In [2], a linear optimal tracking controller was designed, in which the main idea is to simplify the dynamics of the three-wheeled OMR as a linear time invariant model by using the kinematics. In [3], based on a dynamic model without considering motor dynamics, an adaptive motion controller was synthesized via the adaptive backstepping approach. In [4], feedback linearization strategy was used to compensate the static friction, and then a model-predictive control scheme was applied to trajectory tracking control of a three-wheeled OMR. In [5], generalized proportional integral (GPI) observer was employed to design the controller, in which the unmodeled dynamics and nonlinearities, etc., are considered as a perturbation input. In [6], a smooth switching adaptive robust controller was proposed to switch between a nominal adaptive linearizing controller and a deputy adaptive sliding-mode controller. However, these methods actually stem from the well-known control theory, thereby neglecting the natural structure imposed by the physical character of the robot system. One common problem of these methods is that the differential feedback is necessary.

On the other hand, passivity is one of the most fundamental properties of robotic systems [7]. It has been a very powerful concept in many control problems in robotics: stability analysis [8, 9], teleoperation control [10–12], flexible robot control [13–15], to name a few. However, so far, it has been overlooked for the control problem of OMRs.

In this paper, a passivity-based trajectory tracking control system is designed for a three-wheeled OMR with MY wheel-II. The proposed control design is simple to be implemented in practice, because of an effective exploitation of the structure of robot dynamics. The passivity property of the open-loop dynamic system is analyzed based on a dynamic model. We find that the robot is a fully damped system and the damping forces of the robot itself are large enough due to the large gear reduction ratio of motors. Then energy shaping plus damping approach is applied to our robot, wherein only energy shaping is necessary due to enough damping forces of the robot itself. In other words, the disadvantages of differential feedback, such as amplifying the measurement noise, can be avoided. Globally asymptotic stability is guaranteed. Both simulations and experimental results show the effectiveness of the proposed control design.

## Methods

In this section, we first derive a dynamic model for the omnidirectional mobile robot, and then the passivity property of the open-loop robot dynamic system is analyzed.

**Fig. 1:**
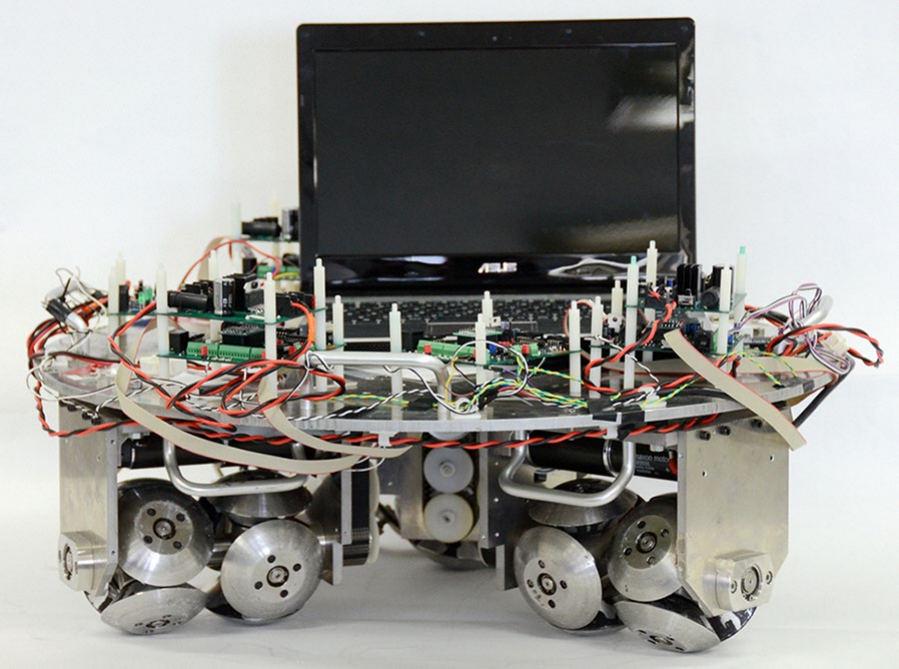
Prototype platform developed by our laboratory

The prototype platform with three MY wheel-II assemblies arranged at 120 degree intervals beneath the steel disk is shown in Fig. 1. Each assembly is actuated with a DC motor. For a detailed description of the MY wheel-II mechanism and the prototype platform, the readers are referred to [16].

**Fig. 2:**
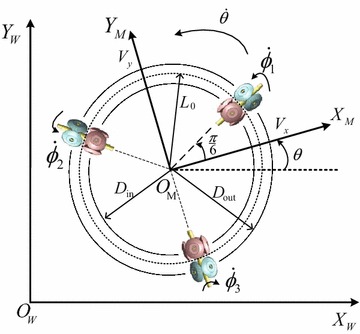
Coordinate frames of the omnidirectional mobile robot

The two coordinate frames used in the modeling are shown in Fig. 2: the world coordinate frame $\left\{ W \right\}$ fixed on the ground and the moving coordinate frame $\left\{ M \right\}$ fixed on the robot geometric center. The nomenclature is defined in Table 1.

**Table 1:** Nomenclature

World coordinate frame	
$${\mathbf {q}} = \left[ {\begin{array}{lll} x &\quad y &\quad \theta \\ \end{array} } \right] ^{\mathrm{T}}$$	Robot position and orientation angle
$${\mathbf{V}}_{\mathrm{M}} = \left[ {\begin{array}{lll} {V_x } &\quad {V_y } &\quad {\dot{\theta } } \\ \end{array}} \right] ^{\mathrm{T}}$$	Robot translational velocity and rotational angular rate
Mechanical constants	
* m*	Robot mass
$I_\mathrm{v}$	Robot moment of inertia around the mass center of the robot
$I_\mathrm{w}$	Wheel moment of inertia around the wheel shaft
* r*	Wheel radius
$D_{\mathrm{in}}$	Inner contact radius
$D_{\mathrm{out}}$	Outer contact radius
$L_{0}$	Average contact radius
$I_0$	Combined moment of inertia of motor, gear train and wheel referred to the motor shaft
$b_0$	Combined viscous friction coefficient of the motor, gear and wheel shaft.
$k_{\mathrm{b}}$	Motor back EMF constant
$k_{\mathrm{t}}$	Motor torque constant
$R_{\mathrm{a}}$	Motor armature resistance
* n*	Gear reduction ratio

Note that, as shown in Figs. 1 and 2, each MY wheel-II assembly has two contact points with the ground, and therefore two contact radiuses exist for each wheel (i.e., $D_{\mathrm{in}}$ and $D_{\mathrm{out}}$) [17]. In our previous work [17], a continuous dynamic model including the DC motor dynamics has been derived for the robot by using an average contact radius (i.e., ${L_0} = {({D_{\mathrm{in}}} + {D_{\mathrm{out}}})}{/}2)$, while the resulting parameter errors are considered as perturbations to the nominal dynamic model. In addition, it is assumed that no slippage is between the wheel and the motion surface. The coulomb and viscous friction, dead-zone and backlash are also unmodeled.

The coordinate transformation matrix from the moving coordinate frame to the world coordinate frame is as follows:

1 $$\begin{aligned} {}_{\text {M}}^{\text {W}} {\mathbf{R}} = \left[{\begin{array}{lll} {\cos \theta } &\quad { - \sin \theta }&\quad 0 \\ {\sin \theta } &\quad {\cos \theta } &\quad 0 \\ 0 &{}\quad 0 &{}\quad 1 \\ \end{array}} \right] . \end{aligned}$$

We get

2 $$\begin{aligned} {\dot{\mathbf{q}}} = {}_{\text {M}}^{\text {W}} {\mathbf{RV}}_{\text {M}}. \end{aligned}$$

The dynamic model of the mobile robot including motor dynamics expressed in the directions of ${X}_{\mathrm{M}}$ and ${Y}_{\mathrm{M}}$ is given as:

3 $$\begin{aligned} {\mathbf{M}}_0 {\dot{\mathbf{V}}}_{\mathrm{M}} + {\mathbf{C}}_0 {\mathbf{V}}_{\mathrm{M}} = {\mathbf{B}}_0 {\mathbf{u}}, \end{aligned}$$

where

$$\begin{aligned} {{\mathbf {M}}_0}&= \frac{1}{{{\beta _2}}}\left[ \begin{array}{lll} {\frac{3}{2}{\beta _0} + m}&{}\quad 0&{}\quad 0 \\ 0&{}\quad {\frac{3}{2}{\beta _0} + m}&{}\quad 0 \\ 0&{}\quad 0&{}\quad {3{\beta _0}{L_0}^2 + {I_v}} \end{array} \right] , \\ {{\mathbf {C}}_0} &= \frac{1}{{{\beta _2}}} \left[ \begin{array}{lll} {\frac{3}{2}{\beta _1}}&{}\quad {{{ - }}m\dot{\theta } }&{}\quad 0 \\ {m\dot{\theta } }&{}\quad {\frac{3}{2}{\beta _1}}&{}\quad 0 \\ 0&{}\quad 0&{}\quad {3{\beta _1}{L_0}^2} \end{array} \right] , \\ {{\mathbf {B}}_0} &= \left[ \begin{array}{lll} { - \frac{1}{2}}&{}\quad { - \frac{1}{2}}&{}\quad 1 \\ {\frac{{\sqrt{3} }}{2}}&{}\quad { - \frac{{\sqrt{3} }}{2}}&{}\quad 0 \\ {{L_0}}&{}\quad {{L_0}}&{}\quad {{L_0}} \end{array} \right] , \end{aligned}$$

${\beta }_0 = \frac{{n^2 I_0 }}{{r^2 }},\,{\beta }_1 = \frac{{n^2 }}{{r^2 }}\left( b_0 + \frac{{k_{\mathrm{t}} k_{\mathrm{b}} }}{{R_{\mathrm{a}} }}\right) ,\,{\beta }_2 = \frac{{nk_{\mathrm{t}} }}{{rR_{\mathrm{a}} }}$. The control input, ${\mathbf {u}} = [u_1 \quad u_2 \quad u_3 ]^{\mathrm{T}}$, is the supplied voltage of three motors.

The robot dynamic model in the world coordinate frame can be obtained by combining (1), (2), (3) [17]:

4 $$\begin{aligned} {{\mathbf {M}}_1}{{\ddot{\mathbf{q}}}} + {{\mathbf {C}}_1}{\dot{\mathbf{q}}} = {{\mathbf {B}}_1}{\mathbf {u}}, \end{aligned}$$

where $${{\mathbf {M}}_1}= {\mathbf{M}}_0 {}_{\text {M}}^{\text {W}} {\mathbf{R}}^{\mathrm{T}},\,{{\mathbf {C}}_1}= {{\mathbf{M}}_0}{\frac{\mathrm{d}}{{\mathrm{d}t}}{}_{\text {M}}^{\text {W}}{{\mathbf{R}}^{\mathrm{T}}}} + {{\mathbf{C}}_0}{}_{\text {M}}^{\text {W}}{{\mathbf{R}}^{\mathrm{T}}},\,{{\mathbf {B}}_1}= {\mathbf{B}}_0$$.

To facilitate the analysis of passivity property, by premultiplying (4) by ${}_{\text {M}}^{\text {W}} {\mathbf{R}}$, we have,

5 $$\begin{aligned} {{\mathbf{M}}}{\ddot{\mathbf{q}}} + {{\mathbf{C}}}{\dot{\mathbf{q}}} + {\mathbf{D}}{\dot{\mathbf{q}}} = {\varvec{\tau }}, \end{aligned}$$

where ${\varvec{\tau }} = {\mathbf {Bu}}$, which is considered as the virtual control input in this paper, and

$$\begin{aligned} {\mathbf {M}}& {}= \frac{1}{{{\beta _2}}}\left[ {\begin{array}{lll} {\frac{3}{2}{\beta _0} + m}&{}\quad 0&{}\quad 0 \\ 0&{}\quad {\frac{3}{2}{\beta _0} + m}&{}\quad 0 \\ 0&{}\quad 0&{}\quad {3{\beta _0}{L_0}^2 + {I_v}} \end{array}} \right] , \\ {\mathbf {C}}& {}= \frac{1}{{{\beta _2}}}\left[ {\begin{array}{lll} 0&{}\quad {\frac{3}{2}{\beta _0}\dot{\theta } }&{}\quad 0 \\ { - \frac{3}{2}{\beta _0}\dot{\theta } }&{}\quad 0&{}\quad 0 \\ 0&{}\quad 0&{}\quad 0 \end{array}} \right] , \\ {\mathbf {D}}& {}= \frac{1}{{{\beta _2}}}\left[ {\begin{array}{lll} {\frac{3}{2}{\beta _1}}&{}\quad 0&{}\quad 0 \\ 0&{}\quad {\frac{3}{2}{\beta _1}}&{}\quad 0 \\ 0&{}\quad 0&{}\quad {3{\beta _1}{L_0}^2} \end{array}} \right] , \\ {{\mathbf {B}}}& {}= \frac{1}{2}\left[ {\begin{array}{lll} { - \cos \theta - \sqrt{3} \sin \theta }&{}\quad { - \cos \theta + \sqrt{3} \sin \theta }&{}\quad {2\cos \theta } \\ { - \sin \theta + \sqrt{3} \cos \theta }&{}\quad { - \sin \theta - \sqrt{3} \cos \theta }&{}\quad {2\sin \theta } \\ {2{L_0}}&{}\quad {2{L_0}}&{}\quad {2{L_0}} \end{array}} \right] , \end{aligned}$$

and ${\mathbf{D}}{\dot{\mathbf{q}}}$ is the dissipative force, due to the combined viscous friction of the motor, gear and wheel shaft, as well as the motor armature resistance. This relates to the loss or dissipation of energy. Moreover, since ${\mathbf {D}}$ is positive definite, the robot is a fully damped system [18].

It can be seen that the inertia matrix ${\mathbf {M}}$ is symmetric, positive definite, and both ${\mathbf {M}}$ and ${{\mathbf {M}}^{ - 1}}$ are uniformly bounded. In addition, the total energy of the open-loop dynamic system (5) is

6 $$\begin{aligned} V({\mathbf{q}},{\dot{\mathbf{q}}}) =\frac{1}{2}{{\dot{\mathbf{q}}}^{\mathrm{T}}}{\mathbf{M}}{\dot{\mathbf{q}}}. \end{aligned}$$

The time derivative of the energy function (6) along (5) is:

$$\begin{aligned} \dot{V}({\mathbf{q}},{\dot{\mathbf{q}}})& = {{{\dot{\mathbf{q}}}}^{\mathrm{T}}}{\mathbf{M}}{\ddot{\mathbf{q}}} \\& = {{{\dot{\mathbf{q}}}}^{\mathrm{T}}}({{\tau }} - {\mathbf{C}}{\dot{\mathbf{q}}} - {\mathbf{D}}{\dot{\mathbf{q}}}) \\& = {{{\dot{\mathbf{q}}}}^{\mathrm{T}}}{{\tau }} - {{{\dot{\mathbf{q}}}}^{\mathrm{T}}}{\mathbf{C}}{\dot{\mathbf{q}}} - {{{\dot{\mathbf{q}}}}^{\mathrm{T}}}{\mathbf{D}}{\dot{\mathbf{q}}} \\& = {{{\dot{\mathbf{q}}}}^{\mathrm{T}}}{{\tau }} - {{{\dot{\mathbf{q}}}}^{\mathrm{T}}}{\mathbf{D}}{\dot{\mathbf{q}}}. \end{aligned}$$

Note that ${\mathbf {D}}$ is symmetric and positive definite and thus ${{\dot{\mathbf{q}}}^{\mathrm{T}}}{\mathbf {D}}{\dot{\mathbf{q}}} > 0$. Therefore, according to the standard passivity definition [19], (5) defines an output strictly passive mapping from the virtual control input ${\varvec{\tau }}$ to ${\dot{\mathbf{q}}}$. Note that, the passive mapping from the real control input ${\mathbf {u}}$ to ${\dot{\mathbf{q}}}$ cannot be guaranteed.

There are two steps in the passivity-based control approach, i.e., energy shaping and damping injection. The first step is an energy shaping stage where the potential energy of the system is modified in such a way that the new potential energy function has a global and unique minimum in the desired equilibriums. Second, a damping injection stage where the dissipation function is modified to ensure global asymptotic stability. For Eq. (5), it is observed that the potential energy is absent. The energy shaping is thus indispensable. However, the damping injection stage can be avoided if the dissipative forces of the robot itself ${\mathbf {D}}{\dot{\mathbf{q}}}$ are large enough to satisfy the control system performance requirements. In other words, by making use of the structure of the robot dynamics, the controller design can become easy and simple. Indeed, it is shown in our simulation and experiments that the controller is able to achieve good performance even though no damping is injected into the system.

### *Remark 1*

The matrix ${\mathbf{D}}$ can be rewritten as follows:

$$\begin{aligned} {\mathbf{D}} = \frac{{n{R_{\mathrm{a}}}}}{{r{k_{\mathrm{t}}}}}\left( {b_0} + \frac{{{k_{\mathrm{t}}}{k_{\mathrm{b}}}}}{{{R_{\mathrm{a}}}}}\right) \left[ {\begin{array}{lll} {\frac{3}{2}}&{}\quad 0&{}\quad 0\\ 0&{}\quad {\frac{3}{2}}&{}\quad 0\\ 0&{}\quad 0&{}\quad {3{L_0}^2} \end{array}} \right] . \end{aligned}$$

It can be seen that the dissipative force ${\mathbf {D}}{\dot{\mathbf{q}}}$ is related with the gear reduction ratio and wheel radius. More specifically, the dissipative force has a positive correlation with the gear reduction ratio *n* and an inverse correlation with the wheel radius *r*.

### *Remark 2*

It is worth pointing out that the robot is a continuous linear dynamic system when the robot moves only with translational motion, and no parameter uncertainties of the contact radius $L_0$ exist in the robot dynamics. However, the parameter uncertainties in the robot contact radius $L_0$ will appear in the robot dynamics if the robot moves with rotational motion. In fact, the robot is an autonomous switched nonlinear system in this case [17].

## Control system design

### Control design

In this section, we derive a trajectory tracking controller only with the energy shaping. The tracking control problem is formulated as follows: Given a reference trajectory $${{\mathbf{q}}_d}(t) = {\left[ {\begin{array}{lll}{{x_d}}&\quad {{y_d}}&\quad {{\theta _d}}\end{array}} \right] ^{\mathrm{T}}}$$, which is bounded and twice continuously differentiable, find a control input ${\mathbf{u}}(t)$ such that the responses of the robot, $${\mathbf{q}}(t) = {\left[ {\begin{array}{lll}x&\quad y&\quad \theta \end{array}} \right] ^{\mathrm{T}}}$$, converges to $${{\mathbf{q}}_d}(t) = {\left[ {\begin{array}{lll}{{x_d}}&\quad {{y_d}}&\quad {{\theta _d}}\end{array}} \right] ^{\mathrm{T}}}$$ for any initial condition.

The proposed controller is inspired from the well-known passivity-based controller, ‘PD+’ controller proposed in [20]. The proposed controller is similar to ‘PD+’ controller, but without damping injection, which is directly given as follows:

7 $$\begin{aligned} {{\tau }} = {\mathbf{M}}{{\ddot{\mathbf{q}}}_d} + ({\mathbf{C}}({\dot{\mathbf{q}}}) + {\mathbf{D}}){{\dot{\mathbf{q}}}_d} - {{\mathbf{K}}_{\mathrm{p}}}{\mathbf{e}}, \end{aligned}$$

where ${{\mathbf{K}}_{\mathrm{p}}}$ is the controller gain and is positive and symmetric.

With the controller (7), the closed-loop error dynamic system is:

8 $$\begin{aligned} {\mathbf{M}}{\ddot{\mathbf{e}}} + ({\mathbf{C}}({\dot{\mathbf{q}}}) + {\mathbf{D}}){\dot{\mathbf{e}}} + {{\mathbf{K}}_{\mathrm{p}}}{\mathbf{e}} = 0. \end{aligned}$$

The energy of the open-loop system (5) is:

9 $$\begin{aligned} {{\mathbf{H}}_0}({\mathbf{q}},{\dot{\mathbf{q}}}) = \frac{1}{2}{{\dot{\mathbf{q}}}^{\mathrm{T}}}{\mathbf{M}\dot{\mathbf{q}}}. \end{aligned}$$

Then choice of the controller (7) actually modifies the original mechanical energy function (9) into:

10 $$\begin{aligned} {{\mathbf{H}}_1}({\mathbf{e}},{\dot{\mathbf{e}}}) = \frac{1}{2}{{\dot{\mathbf{e}}}^{\mathrm{T}}}{\mathbf{M}{\dot{\mathbf{e}}}} + \frac{1}{2}{{\mathbf{e}}^{\mathrm{T}}}{{\mathbf{K}}_{\mathrm{p}}}{\mathbf{e}}. \end{aligned}$$

Note that ${\mathbf{\tau }}$ is the virtual controller, and the final controller ${\mathbf{u}}$ can be derived as follows:

11 $$\begin{aligned} {\mathbf{u}} = {{\mathbf{B}}^{ - 1}}\left( {\mathbf{M}}{{\ddot{\mathbf{q}}}_d} + \left( {\mathbf{C}} + {\mathbf{D}}\right) {{\dot{\mathbf{q}}}_d} - {{\mathbf{K}}_{\mathrm{p}}}{\mathbf{e}}\right) . \end{aligned}$$

It can be seen that only energy shaping is conducted, and thus, only position feedback is used in our proposed controller. The damping injection is not needed. This is because the motor gear reduction ratio of our robot prototype is large ($n = 185.7$) and we find that the damping force of the robot itself ${\mathbf {D}}{\dot{\mathbf{q}}}$ is enough. The calculated results of ${\mathbf{D}}$ of our robot prototype is:

$$\begin{aligned} {\mathbf{D}} = \left[ {\begin{array}{lll} {144.4}&{}\quad {}&{}\quad {}\\ {}&{}\quad {144.4}&{}\quad {}\\ {}&{}\quad {}&{}\quad {10.6} \end{array}} \right] . \end{aligned}$$

In other words, such a damping injection has already been introduced by the robot itself. However, if the motor gear reduction ratio is small, i.e., direct drive motor, it can be calculated that the damping force of the robot itself will be very small and in this case the damping injection will be indispensable. Whether the damping force of the robot itself is large enough depends on the requirements on the control performances.

It is known that differential feedback usually introduces the problem of noise amplification. Therefore, one advantage of the proposed controller is no differential feedback. It is also noted that only the measurement of the rotational velocity ($\dot{\theta }$) is needed in the controller, while the robot translational velocity ($\dot{x}$ and $\dot{y}$) is not used [see (5)]. This is another advantage of the proposed controller. For example, in the well-known computed torque control, the measurement of the robot velocity ${\dot{\mathbf{q}}}$ is indispensable.

In addition, it can be seen that the closed-loop error dynamics (8) does not result in decoupled linear systems. The damping forces of the robot itself are also reserved. These are the main differences from feedback linearization approaches, such as the well-known computed torque control.

#### *Remark 3*

Although there are parameter uncertainties in ${L_0}$ since the real contact radius of each wheel is ${D_{\mathrm{in}}}$ or ${D_{\mathrm{out}}}$, the parameter uncertainties are not considered in the controller design, in order to facilitate the theoretical analysis. It is shown in our experiments that the control system performs well even though the parameter uncertainties appear when the robot moves with rotation.

#### *Remark 4*

For the stabilization control, it can be seen that the proposed controller (7) can be reduced to a very simple proportional feedback controller. That is,

$$\begin{aligned} {\mathbf{\tau }} = - {{\mathbf{B}}^{ - 1}}{{\mathbf{K}}_{\mathrm{p}}}{\mathbf{e}}. \end{aligned}$$

Therefore, the matrix ${\mathbf{M}},\,{\mathbf{C}}$ and ${\mathbf{D}}$ are not used in the controller, and thus, the robot dynamic parameters involved in these matrixes are not necessarily to be known.

### Stability analysis

We choose the energy function as the Lyapunov function:

12 $$\begin{aligned} {\mathbf{V}}({\mathbf{e}},{\dot{\mathbf{e}}}) = \frac{1}{2}{{\dot{\mathbf{e}}}^{\mathrm{T}}}{\mathbf{M}}{\dot{\mathbf{e}}} + \frac{1}{2}{{\mathbf{e}}^{\mathrm{T}}}{{\mathbf{K}}_{\mathrm{p}}}{\mathbf{e}}. \end{aligned}$$

Since ${{\mathbf{K}}_{\mathrm{p}}}$ is positive and symmetric, and ${{\mathbf{C}}}$ is skew-symmetric, then the time derivative of (12) becomes

$$\begin{aligned} {\dot{\mathbf{V}}}({\mathbf{e}},{\dot{\mathbf{e}}})& ={{{\dot{\mathbf{e}}}}^{\mathrm{T}}}{\mathbf{M}}{\ddot{\mathbf{e}}} + {{\mathbf{e}}^{\mathrm{T}}}{{\mathbf{K}}_{\mathrm{p}}}{\dot{\mathbf{e}}}\\ &=- {{{\dot{\mathbf{e}}}}^{\mathrm{T}}}\left( \left( {\mathbf{C}}({\dot{\mathbf{q}}}) + {\mathbf{D}}\right) {\dot{\mathbf{e}}} + {{\mathbf{K}}_{\mathrm{p}}}{\mathbf{e}}\right) + {{\mathbf{e}}^{\mathrm{T}}}{{\mathbf{K}}_{\mathrm{p}}}{\dot{\mathbf{e}}}\\ &=- {{{\dot{\mathbf{e}}}}^{\mathrm{T}}}{\mathbf{D}}{\dot{\mathbf{e}}}, \end{aligned}$$

which is negative semi-definite. However, the LaSalle’s theorem cannot be applied to this case, since LaSalle’s theorem is applicable for autonomous systems. Here, the closed-loop error dynamics (8) is a non-autonomous system since the matrix ${\mathbf{C}}$ is related with $\dot{\theta } (t)$. Instead of using LaSalle’s theorem, the Matrosov theorem can be applied to show that the control system is globally asymptotically stable. The readers are referred to [20] for the detailed proof.

## Simulations

In this section, simulations of the proposed control system are implemented in MATLAB/Simulink. The robot physical parameters used in the proposed control system design are as follows: ${m} = 33$ kg, $I_{{\rm v}} = 1.35$ ${\mathrm{kg}}\,{{\mathrm{m}}^2},\,R = 0.06$ m, $D_{\mathrm{in}} = 0.147$ m, $D_{\mathrm{out}} = 0.236$ m, $I_0 = 3.15\times 10^{-5}\,{\mathrm{kg}}\,{\mathrm{m}^2},\,k_{\mathrm{t}} = 0.0292\, {\mathrm{N}}\,{\mathrm{{m}/A}},\,k_{\mathrm{b}} = 328$ rpm/V, $n = 185.7,\,b_0 = 1.04 \times 10^{ - 4}\,\,\mathrm{N\,ms/rad},\,R_{\mathrm{a}} = 0.61\,\Omega$.

To verify the effectiveness of the proposed control system design, the robot was commanded to track a circle of 0.8 m radius within 30 s, i.e., ${x_d}(t) = 0.8\cos (\frac{{\pi t}}{{15}})$ m; ${x_d}(t) = 0.8\sin \left( \frac{{\pi t}}{{15}}\right)$ m. The robot initial posture is set as $$\left[ {\begin{array}{*{20}c} {0.8{} {} \text {(m)}} &{} {0{} {} \text {(m)}} &{} {0{} {} \text {(rad)}} \\ \end{array} } \right] ^{\mathrm{T}}$$. In the first 10 s, the robot performs translational motion without rotation, i.e., $\theta _d=0$ rad. After 10 s, the desired robot orientation angle is set as ${\theta _d} = 0.32(t-10)$ rad. The controller gain is set as:

$$\begin{aligned} {{\mathbf{K}}_{\mathrm{p}}} = \left[ {\begin{array}{lll} {20}&{}\quad {}&{}\quad {}\\ {}&{}\quad {20}&{}\quad {}\\ {}&{}\quad {}&{}\quad 20 \end{array}} \right] . \end{aligned}$$

**Fig. 3:**
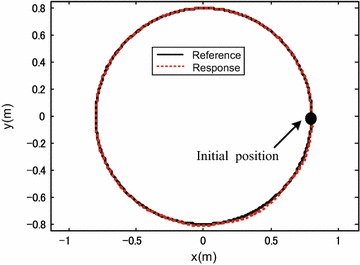
Simulation results: trajectory tracking performance of translational motion

**Fig. 4:**
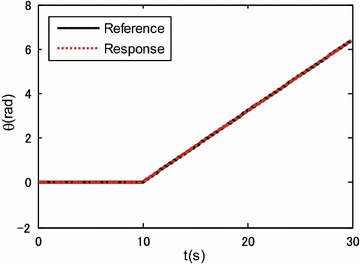
Simulation results: trajectory tracking performance in the orientation direction

**Fig. 5:**
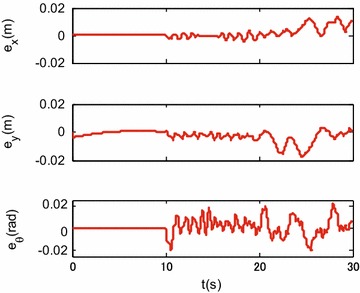
Simulation results: translational and rotational tracking errors

**Fig. 6:**
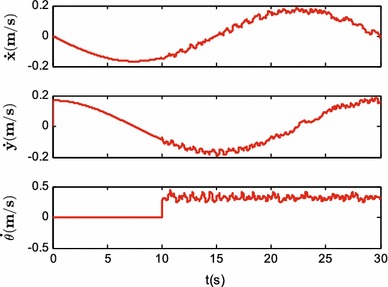
Simulation results: robot velocity

Simulation results are shown in Figs. 3, 4, 5 and 6.

**Fig. 7:**
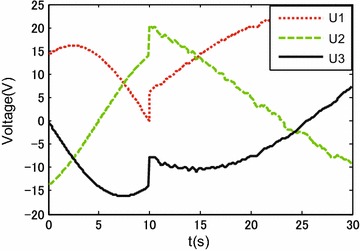
Simulation results: control input ${\mathbf {u}}$, i.e., supplied voltages of three DC motors

Figures 3, 4 and 5 show the tracking performance of the proposed controller. It can be seen that the controller achieves good performance only with position feedback while without velocity feedback. It is also observed that the tracking errors in the first 10 s are near zero, while the tracking errors increase when the robot moves with rotation. As already mentioned before, the robot is a linear system when the robot moves only with translational motion. However, if the robot moves with rotational motion, then the parameter uncertainties in the contact radius $L_0$ take effects on the robot dynamics. In other words, modeling errors resulting from the parameter uncertainties in $L_0$ are introduced into the closed-loop error dynamics and thus the controller performances are reduced. It is also shown in Fig. 6 that the fluctuations in the robot velocity are introduced when the robot moves with rotational motion. This is caused by the parameter uncertainties in $L_0$ and the fact that the parameter uncertainties are not effectively compensated in the controller. In addition, the real control input is shown in Fig. 7.

## Experiments

In this section, we first give a brief introduction of the experimental setup and then present the experimental results. The parameter uncertainties, the robot velocity-related viscous and coulomb friction and nonlinearities (e.g., dead-zone and backlash) are not involved in the derived dynamic model (5). Thus, the effectiveness of the proposed controller should be verified through experiments.

### Experimental setup

**Fig. 8:**
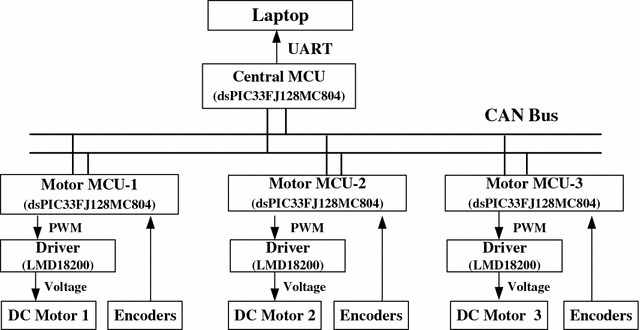
Schematic of the experimental setup

The robot prototype developed in our laboratory is shown in Fig. 1. The complete schematic of the experimental setup is shown in Fig. 8. Experimental data are transmitted to a laptop from the central controller. The communication between the central MCU and the three motor MCUs is via CAN bus, which was programmed to operate at 1 Mb/s. All of the MCUs (dsPIC33FJ128MC804 from Microchip, USA) were programmed to operate at 40 million instructions per second. The three DC motors (order number: 323890, Maxon) are identical with gear reduction ratio of 185.7 and nominal voltage of 24 V. An incremental encoder (order number: 225787, Maxon) is installed inside each motor. The three DC motor drivers are identical (LMD18200 from Texas Instruments, USA). Three absolute encoders (MAB2510HS5VSER from MegaMotive, Germany) are used to detect the contact radius. Communication between the central controller and the laptop is via UART, which was programmed to operate at a data transfer rate to 2.5 Mbaud. The robot posture is determined using odometry which is commonly used in the tracking control study of mobile robots [21–23], to name a few. Experimental data are transmitted to a laptop from the central controller.

Finally, the control law was implemented in *C* on the central controller. The sampling time of the control system was set as 10 ms. The Euler’s method was used as the discretization method. The same circle trajectory used in the simulation is selected as the reference trajectory in the experiment. For comparison, the controller gain is set as the same with the simulations, i.e.,

$$\begin{aligned} {{\mathbf{K}}_{\mathrm{p}}} = \left[ \begin{array}{lll} {20}&{}\quad {}&{}\quad {}\\ {}&{}\quad {20}&{}\quad {}\\ {}&{}\quad {}&{}\quad 20 \end{array} \right] . \end{aligned}$$

### Experimental results

**Fig. 9:**
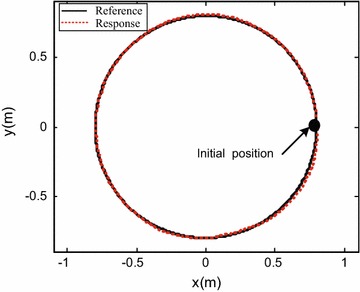
Experimental results: trajectory tracking performance of translational motion

**Fig. 10:**
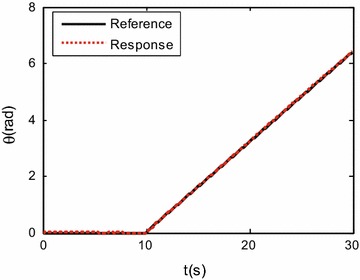
Experimental results: trajectory tracking performance in the orientation direction

**Fig. 11:**
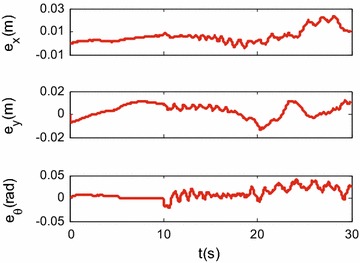
Experimental results: translational and rotational tracking errors

**Fig. 12:**
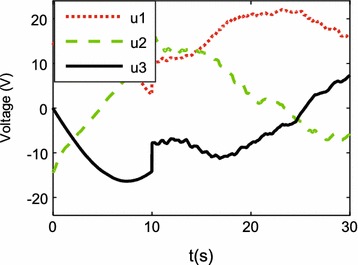
Experimental results: control input ${\mathbf {u}}$, i.e., supplied voltages of three DC motors

Figures 9, 10 and 11 show the tracking performances of the proposed controller. It is observed that the controller achieves good performance with only position feedback. The upper bounds of the translational and rotational tracking errors are about 0.03 m and 0.05 rad, respectively, which are larger than simulation results, respectively. This is because the modeling errors in practice, such as the unmodeled friction forces and estimated parameter values. Figure 12 shows the control input for the three motors. It is shown in both Figs. 11 and 12 that, in the first 10 s, the tracking errors and the control inputs are smooth. However, from 10 to 30 s, fluctuations appear in both the tracking errors and control inputs. This is because, as already mentioned, the parameter uncertainties in the contact radius $L_0$ take effects on the robot dynamics only when the robot moves with rotational motion.

## Conclusions

In this paper, a passivity-based trajectory tracking control has been proposed for an omnidirectional mobile robot. The passivity properties of the prototype robot have been analyzed. It is shown in our analysis that the prototype robot itself is an output strictly passive system and is a fully damped system. The robot itself has enough damping forces due to the large gear reduction ratio of the motors. As a result, only energy shaping (i.e., position feedback) is needed in our proposed controller. In fact, only the rotational velocity of the robot is needed. Stability analysis shows that globally asymptotic stability can be guaranteed. Both simulations and experimental results have shown the effectiveness of the proposed control design.

In the future work, we will improve the performance of the proposed control design by compensating the modeling errors and external disturbances.
